# The staging performance of a modified tumor-node-metastasis staging system incorporated with lymphovascular invasion in patients with esophageal squamous cell carcinoma

**DOI:** 10.3389/fonc.2022.1018827

**Published:** 2022-10-13

**Authors:** Weitao Zhuang, Hansheng Wu, Rixin Chen, Xiaosong Ben, Shujie Huang, Zihao Zhou, Junhan Wu, Yong Tang, Guibin Qiao

**Affiliations:** ^1^ Department of Thoracic Surgery, Guangdong Provincial People’s Hospital, Guangdong Academy of Medical Sciences, Guangzhou, China; ^2^ Department of Thoracic Surgery, The First Affiliated Hospital of Shantou University Medical College, Shantou, China; ^3^ Research Center of Medical Sciences, Guangdong Provincial People’s Hospital, Guangdong Academy of Medical Sciences, Guangzhou, China; ^4^ Shantou University Medical College, Shantou, China

**Keywords:** esophageal cancer, lymphovascular invasion, staging, prognosis, pathology

## Abstract

**Background:**

Lymphovascular invasion (LVI) is recognized as an unfavorable prognostic factor for many solid tumors. However, its staging value has not been adequately illustrated in esophageal squamous cell carcinoma (ESCC).

**Methods:**

The clinicopathologic relevance and prognostic impact of LVI were retrospectively analyzed in 822 patients with surgically treated ESCC. Univariate and multivariate analyses were used to determine the independent prognostic factors. Subgroup analyses stratified by pathological stages, nodal status and invasive depth were conducted using Kaplan–Meier method and log-rank test. Multiple staging models based on overall survival (OS) were constructed using Cox regression and evaluated by Harrell’s concordance index (C-index), integrated discrimination improvement (IDI), and net reclassification index (NRI).

**Results:**

LVI was detected in 24.6% of ESCC patients, and its prevalence increased with a higher pathological stage (p < 0.001). In multivariate analysis, LVI was found to be an independent prognostic factor for OS [Hazard ratio (HR) = 1.545, 95% CI, 1.201–1.986), and was associated with unfavorable outcomes in stage I to III ESCC, regardless of nodal status and invasive depth. The staging model that incorporated LVI as an independent factor achieved the greatest improvement in accuracy (ΔC-index: 2.9%), and the greatest added value (IDI 2.8%, p < 0.01; NRI 13.7%, p < 0.05) for prediction of OS in ESCC patients.

**Conclusions:**

LVI can facilitate further survival stratification in ESCC patients. The adoption of LVI as an independent staging factor in the current cancer staging system should be considered and further validated.

## Introduction

Esophageal cancer is one of the leading causes of cancer-related-death worldwide (1). It is characterized by extensive treatment requirements, considerable decrease in quality of life, and unfavorable clinical outcomes (2). Advancement in multidisciplinary therapeutic approaches has gradually improved its prognosis and survivability (2, 3). However, an optimal and individualized treatment usually requires accurate pathological staging and prognostication of patients. The tumor–node–metastasis (TNM) classification system has been continuously updated by the American Joint Committee on Cancer (AJCC) for better prognostication and determination of treatment strategies (4, 5). Staging factors including grade and location of esophageal cancer were once incorporated into the practice of TNM classification system (4, 5); however, their prognostic significance remains controversial (6–10). Therefore, the identification and validation of more sensitive biological factors are essential to further subclassify the patients and facilitate individualized therapeutic strategies (11).

Lymphovascular invasion (LVI), an aggressive tumor histopathological feature, might increase the risk of lymphatic or hematogenous micrometastases for localized diseases (12). LVI is defined as a cluster of neoplastic cells within the lumen of lymphatic or blood vessels on tissue slides (13), and can be easily and reliably assessed microscopically. The adverse prognostic effect of LVI in various solid tumors, including those of lung cancer (14–16), breast cancer (17, 18), urothelial cancer (12, 19), gastric cancer (20), and esophageal squamous cell carcinoma (ESCC) (13, 21–25), has been evaluated and validated by many well-conducted retrospective studies. LVI was proposed as a supplementary factor for pathological nodal (pN) staging system in two prior large sample studies of ESCC (3, 22); however, only the risk of lymphatic metastasis was explored. Other studies were usually limited by their small sample sizes and thus heterogeneity of patients (23, 26), or by the inclusion of only early stage diseases (21, 24, 25). Thus, the practical value of LVI in inadequately examined.

We therefore conducted a retrospective study to characterize the prognostic effect of LVI in ESCC patients with different pathological stages, nodal status, and invasive depth. Predictive models were also constructed to evaluate the predictive accuracy and to investigate the potential utilization of LVI as an independent staging factor in ESCC patients.

## Methods

### Study population

A study cohort was identified from an ESCC database, which included 1,265 consecutive cases from January 2009 to October 2019 in Guangdong Provincial People’s Hospital, China. Information in this database has been maintained by a regular collection and review of the sociodemographic and clinicopathologic data from electronic medical records (EMR), and the follow-up information of individual patients was collected mostly through phone call or in our outpatient clinic. The selection criteria for patient enrollment included: (i) pathologically confirmed ESCC; (ii) surgically treated ESCC; (iii) pathological stage I to stage IVA; (iv) aged 18–80 years old; (v) no major postoperative complications such as respiratory clinicopathological information was retrieved from the ESCC database by one researcher and confirmed in the EMR system by another. Surgically treated patients who had no description of LVI status in their pathological reports were excluded from the statistical analysis (n=52). Finally, 822 patients were eligible for retrospective analysis (see **Supplementary Figure S1**). This study was approved by the Institutional Review Board of Guangdong Provincial People’s Hospital, with patient informed consent waived (No. GDREC2019687H).

### Preoperative evaluation and surgical procedures

All patients had an Eastern Cooperative Oncology Group (ECOG) performance status score of 0–2 and had routinely undergone preoperative workup, including esophageal endoscopy, barium swallow, cardiopulmonary function evaluation, computed tomography of chest and abdomen, as well as biochemical profile of blood. Endoscopic ultrasound or whole-body flurodeoxyglucose positron emission tomography/computed tomography (FDG-PET/CT) were offered to selected patients only. The surgical types included transthoracic open esophagectomy (open procedure), hybrid minimally invasive esophagectomy (hybrid procedure), or totally minimally invasive esophagectomy (TMI procedure). Surgical approaches included thoracotomy, Ivor Lewis esophagectomy and lymphadenectomy was performed in patients with a curative intent.

### Histopathologic assessment

The pathological evaluation of all resected specimens was performed by one pathologist and confirmed by another according to the institutional diagnostic protocol. Differentiation between lymphatic or vascular invasion using immunohistochemical (IHC) staining was not required in our institution. Pathological reports with typical hematoxylin and eosin (H&E) stained slides on EMR were reassessed by two researchers independently. Any disagreement on LVI status was resolved by reexamining the stored paraffin-embedded specimens in the archive room. Pathological staging of all the patients was reassessed following the 8th edition of the AJCC TNM staging system (5).

### Neoadjuvant and adjuvant therapies

Platinum-based neoadjuvant or adjuvant chemotherapy or chemoradiotherapy was recommended for selected patients according to the National Comprehensive Cancer Network (NCCN) guideline for esophageal cancer. Chemotherapy regimens usually included platinum plus docetaxel or 5-fluorouracil. Most patients had received at least two cycles of neoadjuvant chemotherapy before assessing the eligibility for surgery.

### Patient follow-up

The patients were followed up at our outpatient clinics every 3 months after esophagectomy for the first 2 years and every 6 months for the following 3 years. Follow-up examinations included serum tumor biomarkers, chest radiography, thoracic and abdominal CT scans, abdominal ultrasound, bone scan, or a cranial MRI scan for the surveillance of recurrence or metastasis. Patients who did not comply with the follow-up plan were contacted regularly *via* telephone to renew their vital status. If the patient died, the date of death or any information on cancer recurrence would be collected from their family members. Patients who were lost to follow-up or still alive after the cut-off date for follow-up were classified as censored. Overall survival (OS) was defined as the time from esophagectomy to the date of death. The patient follow-up was cut off on March 31, 2020 for the final data analysis, and the median follow-up time was 53.9 months (95% CI, 50.5–57.4 months).

### Statistical analysis

The categorical and ordinal variables between the groups with or without LVI were compared with the Chi-square test and Mann-Whitney U test, respectively. Data not recorded in EMR were treated as missing values in the statistical analysis. Survival curves were depicted using Kaplan-Meier method and compared by the log-rank test. A univariate Cox regression analysis was conducted with its significance level cross-validated by log-rank test. All covariates with a significance level of p < 0.15 in the univariate analysis were included in the multivariate analysis using the Cox proportional hazards model. Outcome prediction models combining different independent prognostic factors were generated using Cox regression with 1000-bootstrap resampling and compared by C-index, with a larger value indicating better predictive accuracy. The C-index was calculated using R software version 3.6.3 (R Foundation for Statistical Computing, Vienna, Austria) with the “rms” and “survcomp” packages. Subsequently, the “survIDINRI” package (https://cran.r-project.org/web/packages/survIDINRI/index.html) was used for the calculation of integrated discrimination improvement (IDI) and net reclassification index (NRI) of different modified staging systems, with 1,000 iterations for each calculation. The p-value of IDI and NRI was obtained through Z statistics (27). The cut-off point for the NRI calculation was set as 5 years. IDI and NRI can help to quantify the net added value contributed by LVI, and they represent the new metrics to supplement the C-index in the model assessment. NRI is more sensitive than C-index and easier to understand. However, NRI fails to evaluate the overall improvement across different time points, which can be overcome by calculating the IDI (28). If NRI or IDI >0, this indicates that the new model has a positive improvement of predictive value compared to the original one. A larger difference means that the new model is better. The calculation method and interpretation of IDI and NRI have been described in detail in previous studies (28, 29). Other statistical analyses were performed using SPSS 23.0 software for Windows (IBM Corp., Armonk, NY, USA), and a two-sided p value < 0.05 was considered statistically significant.

## Results

### Patient characteristics

The demographic and clinicopathologic characteristics of the 822 patients are summarized in **Table 1**. LVI was found in 24.6% of all patients (202 out of 822) and 22.0% of these patients receiving neoadjuvant therapies (24 out of 109), which was similar to the results of previous studies (24.6%–33.8%) (13, 22, 23). Patients with a more advanced pathological stage (p < 0.001) were found to have a higher prevalence of LVI. Interestingly, LVI was not associated with the histological differentiation of ESCC (p = 0.355). Otherwise, the LVI-positive and LVI-negative groups were similar regarding age, sex, body mass index (BMI), Charlson comorbidity index (CCI) and surgical approaches, with all p values > 0.05 (**Table 1**).

**Table 1:** Association of lymphovascular invasion with clinicopathologic characteristics of patients with resected esophageal squamous cell carcinoma.

Characteristics		LVI negative (n = 620) (%)	LVI positive (n = 202) (%)	p value
**Age (years)**	≤ 60	286 (46.1)	110 (54.5)	0.060†
	> 60	334 (53.9)	92 (45.5)	
**Sex**	Male	495 (79.8)	163 (80.7)	0.816†
	Female	125 (20.2)	39 (19.3)	
**BMI (kg/m2) ¶**	< 18.5	83 (14.8)	27 (14.8)	0.590‡
	18.5-23.9	297 (53.1)	93 (50.8)	
	> 23.9	179 (32.1)	63 (34.4)	
**CCI**	0-1	571 (92.1)	187 (92.6)	0.826‡
	≥ 2	49 (7.9)	15 (7.4)	
**Tumor location**	Upper	76 (12.2)	24 (11.9)	0.98†
	Middle	419 (67.1)	135 (66.8)	
	Lower	129 (20.7)	43 (21.3)	
**Pathological stage**	IA+IB	84 (13.5)	14 (6.9)	< 0.001‡
	IIA+IIB	320 (51.6)	58 (28.7)	
	IIIA+IIIB	178 (28.7)	87 (43.1)	
	IVA	38 (6.1)	43 (21.3)	
**Pathological T stage**	pT1a+1b	92 (14.8)	12 (5.9)	< 0.001‡
	pT2	171 (27.6)	38 (18.8)	
	pT3	354 (57.1)	148 (73.3)	
	pT4a+4b	4 (0.5)	4 (2.0)	
**Pathological N stage**	pN0	388 (62.6)	74 (36.6)	< 0.001‡
	pN1	136 (21.9)	44 (21.8)	
	pN2	62 (10.0)	42 (20.8)	
	pN3	34 (5.5)	42 (20.8)	
**Tumor grade**	G1	85 (13.7)	22 (10.9)	0.355‡
	G2	404 (65.2)	134 (66.3)	
	G3	131 (21.1)	46 (22.8)	
**PNI ¶**	Yes	380 (65.3)	109 (58.0)	0.070†
	No	202 (34.7)	70 (42.0)	
**Surgical type**	Open	108 (17.4)	34 (16.8)	0.904†
	Hybrid	337 (54.3)	107 (53.0)	
	TMI	175 (28.2)	61(30.2)	
**Surgical approach**	Thoracotomy	69 (11.1)	19 (9.4)	0.331†
	Ivor-Lewis	88 (14.2)	39 (19.3)	
	McKeown	463 (74.7)	144 (71.3)	
**Neoadjuvant therapy**	Yes	85 (13.8)	24 (11.8)	0.463†
	No/Unknown	529 (86.2)	179 (88.2)	
**Adjuvant therapy**	Yes	210 (33.9)	103 (51.0)	< 0.001†
	No/Unknown	410 (66.1)	99 (49.0)	

LVI, lymphovascular invasion; BMI, body mass index; CCI, Charlson comorbidity index; PNI, perineural invasion. TMI, totally minimally invasive. † Chi-square test, ‡ Mann–Whitney U test. ¶ Missing data: BMI (n = 80), PNI (n = 61).

### Prognostic importance of LVI

In the overall study cohort, the 5-year OS rate was significantly lower in patients with LVI than in patients without LVI (36.9% vs. 58.5%, p < 0.001). Among the 109 patients receiving neoadjuvant therapies, a similar trend of unfavorable survival outcome was observed in the LVI-positive group (median OS: 60.93 months vs. 27.67 months, p=0.106), albeit not reaching a level of statistically significance (see **Supplementary Figure S2**). The univariate analysis suggested that sex, BMI, tumor grade, pathological T or N classification, perineural invasion (PNI), LVI, administration of neoadjuvant or adjuvant therapies were the potential prognostic candidates of ESCC (all p < 0.15, see **Supplementary Table S1**). In the multivariate analysis adjusted for the abovementioned covariates (**Table 2**), LVI was found to be an independent prognostic factors of OS [hazard ratio (HR) = 1.561, 95% CI, 1.214–2.007, p = 0.001].

**Table 2:** Multivariate Cox regression analysis for overall survival in patients with resected esophageal squamous cell carcinoma.

Variables (Ref.) †	Adjusted Hazard Ratio	95% Confidence Interval		p value
		Upper limit	Lower limit	
**Sex (Male)**				0.541
Female	0.910	0.673	1.231	
**BMI (< 18.5)**				0.482
18.5-23.9	0.999	0.718	1.391	
> 23.9	0.853	0.592	1.230	
**Pathological T Stage (pT1a+1b)**				0.005
pT2	1.376	0.797	2.375	
pT3	2.073	1.247	3.447	
pT4a+4b	2.307	0.655	8.129	
**Pathological N stage (pN0)**				0.003
pN1	1.517	1.119	2.057	
pN2	1.265	0.886	1.805	
pN3	1.897	1.303	2.763	
**Tumor Grade (G1)**				0.084
G2	1.385	0.957	2.005	
G3+4	1.612	1.059	2.453	
**PNI (No)**				0.298
Yes	1.136	0.894	1.443	
**LVI (No)**				0.001
Yes	1.561	1.214	2.007	
**Neoadjuvant therapy (No/Unknown)**				0.257
Yes	1.219	0.866	1.717	
**Adjuvant therapy (No/Unknown)**				0.635
Yes	0.940	0.728	1.214	

Ref., reference; BMI, body mass index; PNI, perineural invasion; LVI, lymphovascular invasion. † All variables had a p < 0.15 (log-rank test) in univariate analysis.

### Subgroup analysis stratified by pathological stages

**Figure 1:**
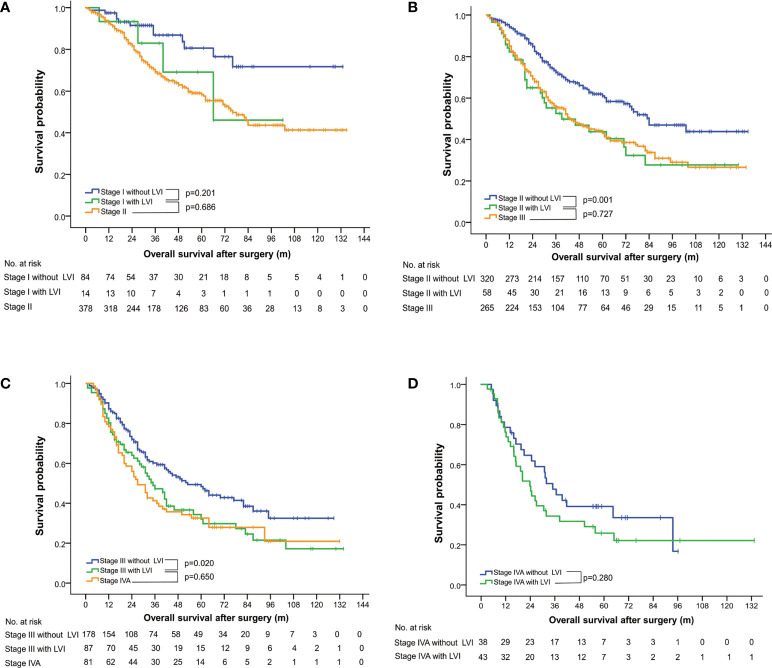
Overall survival curves stratified by status of lymphovascular invasion and pathological stages, based on the 8th edition of the AJCC TNM classification system for esophageal squamous cell carcinoma. LVI, lymphovascular invasion.

A subgroup analysis was conducted to investigate the staging value of LVI based on the 8th edition of the AJCC TNM staging system (5). The OS among patients with a pathological stage I to III could be further subclassified by LVI status (**Figure 1**). LVI-positive patients with lower pathological stages were found to consistently achieve a similar survivorship as the patients with one stage higher (**Figure 1**, **Table 3**). For example, the 5-year OS among patients with LVI-positive stage II ESCC was 43.77%, versus 43.00% in stage III diseases (p = 0.755). Similar results were obtained when comparing the OS among patients of LVI-positive stage I with stage II (p = 0.673), and when comparing LVI-positive stage III with stage IV diseases (p = 0.585).

**Table 3:** Subgroup analysis of overall survival stratified by lymphovascular invasion, pathological stages, N classifications and T classifications.

Group	5-year OS (%)	Median OS (months)	95% CI (months)		p value†	p value†
			Lower	Upper		
Stage I without LVI	80.59	NR	NR		]0.206
Stage I with LVI	69.14	66.70	27.72	105.68		]0.673
Stage II	58.34	76.50	62.54	89.46		
Stage II without LVI	61.08	83.80	62.18	104.42	]0.002
Stage II with LVI	43.77	38.80	11.04	66.56		]0.755
Stage III	43.00	43.20	32.20	54.13		
Stage III without LVI	48.38	52.30	35.54	69.12	]0.023
Stage III with LVI	32.05	35.20	25.85	44.60		]0.585
Stage IVA	32.56	26.40	18.84	33.90		
Stage IVA without LVI	33.56	35.00	26.61	43.33	]0.280
Stage IVA with LVI	25.84	23.80	17.12	30.49		
pT_any_N0 without LVI	64.37	104.30	NR		]0.001
pT_any_N0 with LVI	49.50	53.10	25.78	80.35		]0.720
pT_any_N1	44.49	45.70	26.33	65.13		
pT_any_N1 without LVI	50.15	53.60	35.07	72.19	]0.115
pT_any_N1 with LVI	27.42	32.60	24.55	40.71		]0.190
pT_any_N2	43.28	41.80	22.79	60.88		
pT_any_N2 without LVI	48.22	60.80	35.87	85.80	]0.070
pT_any_N2 with LVI	32.18	36.30	22.69	49.91		]0.523
pT_any_N3	30.22	26.40	16.28	36.46		
pT_any_N3 without LVI	NR	35.00	27.12	42.82	]0.259
pT_any_N3 with LVI	24.44	23.80	17.21	30.40		
pT1N_any_ without LVI	76.10	NR	NR		]0.869
pT1N_any_ with LVI	58.30	66.70	10.47	122.93		]0.406
pT2N_any_	61.40	NR	NR			
pT2N_any_ without LVI	67.79	NR	NR		]0.005
pT2N_any_ with LVI	36.92	42.00	25.63	58.38		]0.835
pT3N_any_	44.96	49.10	39.04	59.16		
pT3N_any_ without LVI	50.33	60.90	48.82	73.04	]< 0.001
pT3N_any_ with LVI	32.59	30.60	23.67	37.53		]0.984
pT4N_any_	20.00	31.90	10.15	53.72		

T, tumor; T_any_, pathological stage T1-T4; N, node; N_any_, pathological stage N0-N3; LVI, lymphovascular invasion; OS, overall survival; 95% CI, 95% confidence interval; NR, not reached. †Log-rank test.

### Subgroup analysis stratified by nodal status

**Figure 2:**
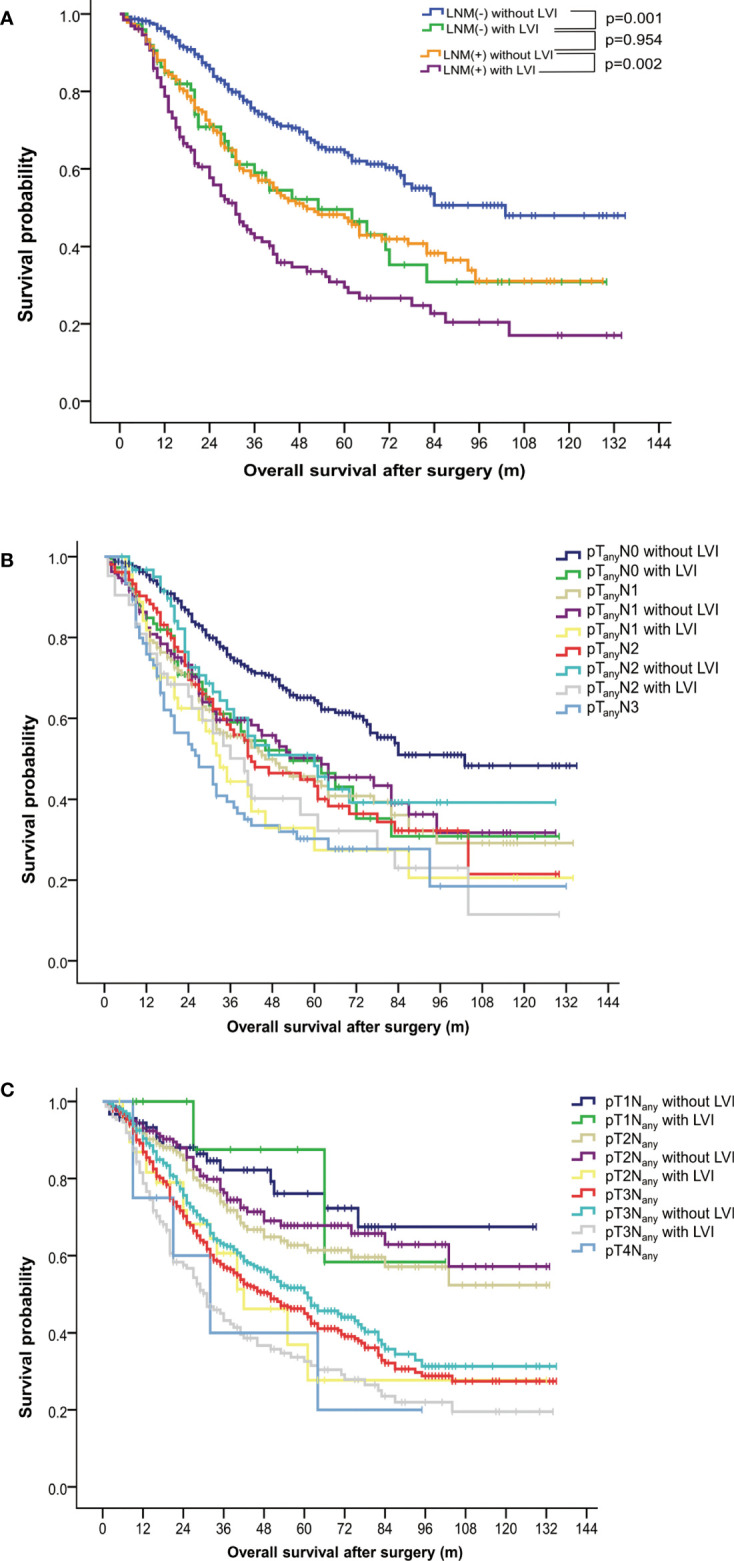
Subgroup analysis of overall survival in patients with esophageal squamous cell carcinoma. **(A)** Survival curves stratified by status of lymphovascular invasion and lymph node metastasis. **(B)** Survival curves stratified by status of lymphovascular invasion and pathological N classifications. **(C)** Survival curves stratified by status of lymphovascular invasion and pathological T classifications. LNM, lymph node metastasis; LVI, lymphovascular invasion.

In the LNM-negative group, 16.0% (74 out of 462) of patients had LVI. In contrast, 35.6% (128 out of 360) of LNM-positive patients were found to have LVI (**Table 1**). Survival analysis of both groups suggested an unfavorable outcome of positive-LVI regardless of nodal status (**Figure 2A**). The 5-year OS rates in LNM-negative diseases with or without LVI were 49.5% versus 64.2% (p = 0.001). Similarly, the 5-year OS rates in LNM-positive diseases with or without LVI were 29.4% versus 47.3% (p = 0.002). It is worth noting that the LNM-negative diseases with LVI had demonstrated a similar survival outcome to LNM-positive diseases without LVI (OS: p = 0.954) (**Figure 2A**). However, further analysis of OS on pathological N classifications did not distinguish the subgrouping value of LVI among patients with N1 (p = 0.115), N2 (p = 0.070) and N3 (p = 0.259) diseases (**Table 3**, **Figure 2B**).

### Subgroup analysis stratified by invasive depth

The 5-year OS rates among patients with and without LVI were 58.30% versus 76.10% (p = 0.869), 36.92% versus 67.79% (p = 0.005), 32.59% versus 50.33% (p < 0.001) in pT1, pT2, and pT3 ESCC, respectively. ESCC with positive-LVI also demonstrated an inferior OS comparable to those with a higher pT stage (with all p > 0.05), which was also similar to the findings in a subgroup analysis of the pathological stage (**Table 3**, **Figure 2C**).

### Accuracy and improvement of prognostic prediction models incorporating LVI status

To further determine the staging value of LVI, prognostic prediction models were generated using Cox regression with 1000-bootstrap resampling. The involvement of LVI status as an independent predictive variable in the TNM system (Model II) increased the C-index by 2.9% for OS (**Table 4**), which was superior to the direct upstaging of TNM stage (Model III, increased by 1.6%) or modification of T classification (Model IV, increased by 1.8%) and N classification (Model V, increased by 2.0%). The IDI and NRI demonstrated that Model II (OS: IDI 2.8%, p < 0.01; NRI 13.7%, p < 0.05) provided the greatest net improvement to the original TNM staging system in the prediction of OS compared with Model III, Model IV and Model V (**Table 4**). Internal validation of Model II by the bootstrap method supported LVI as a predictor independent of the current TNM staging system (p = 0.001, see **Supplementary Table S2**).

**Table 4:** Assessment of accuracy and improvement of clinical outcome of different predictive models based on overall survival.

Model	C-index (95% CI)	Bias corrected C-index	IDI	NRI¶
			(95% CI)	(95% CI)
I: Original TNM classification	0.616 (0.587–0.646)	0.616	Ref.	Ref.
II: G+T+N+LVI classification	0.649 (0.617–0.681)	0.645	0.028 (0.006–0.061) **	0.137 (0.020–0.227) *
III: Upstaging of TNM classification †	0.633 (0.604–0.663)	0.633	0.014 (-0.003–0.031)	0.082 (0–0.192) *
IV: Upstaging of T classification ‡	0.635 (0.605–0.665)	0.635	0.011 (-0.010–0.035)	0.043 (-0.057–0.147)
V: Upstaging of N classification ‖	0.637 (0.607–0.666)	0.637	0.014 (-0.004–0.035)	0.030 (-0.105–0.178)

C-index, Harrell’s concordance index. 95% CI, 95% confidence interval. NRI, net reclassification index; IDI, integrated discrimination improvement; Ref., Reference; LVI, lymphovascular invasion. T, tumor. N, node. G, grade. † TNM Stage I, II, III of ESCC with positive-LVI were upgraded to stage II, III, and IV, respectively, and stage IV disease remained at the same stage.

‡ Pathological stage T1, T2, and T3 were upgraded to T2, T3, and T4 in ESCC with positive-LVI, respectively, and T4 disease remained at the same stage.

‖ Pathological stage N0, N1, and N2 were upgraded to N1, N2, and N3 in ESCC with positive-LVI, respectively, and N3 disease remained at the same stage.

All restaging were based on 8th AJCC TNM system.

¶ Cut-off point for NRI calculation was set as 5 years.

*p < 0.05, **p < 0.01, by Z-statistics

## Discussion

Lymphatic and hematogenous metastasis are two of the most critical mechanisms of locoregional and distant recurrence of cancer (27). Although they have been well characterized, the microscopic events of cancer metastasis have not been fully understood (28). LVI appears to be an early microscopic feature to predict regional or distant recurrence in various solid tumors (29–32). Nevertheless, its clinical significance is usually underestimated in ESCC.

In the current study, a thorough analysis of the large-scale ESCC database was performed to investigate the practical significance of LVI regarding prognostic grouping and pathological staging. The association between a more advanced stage of ESCC and a higher prevalence of LVI in our study might suggest its aggressive biological feature. In the multivariate analysis, LVI was found to be an independent predictor of survival outcome, which was in parallel to pathological T and N parameters (**Table 2**). In contrast, tumor grade, a controversial staging factor (6–10), was not an independent prognostic factor in our study. This indicates the higher sensitivity of LVI in prognostication and supports its possible use as a staging factor in resectable ESCC.

A thorough subgroup analysis regarding OS further validated the prognostic grouping value of LVI among ESCC patients in different pathological stages and T classifications, except in patients with stage IV diseases (**Table 2**, **Figure 1**). Interestingly, LVI was found to be an unfavorable factor for OS not only in LNM-negative ESCC, but also in LNM-positive ones (**Figure 2A**), which was in accordance to the findings of a previous large-scale study (22), but in conflict with the results of the other one (13). The results of our study might imply the underlying risks of hematogenous metastasis in LVI-positive patients, which was not discussed in the aforementioned study (13). A simultaneous lymphatic and vascular invasion had been found to associate with a poorer prognosis than lymphatic or vascular invasion alone (33). Therefore, differentiation between lymphatic and vascular invasion by immunohistochemical staining may facilitate further subclassification of LNM-positive patients.

Given the significant prognostic value of LVI, several researchers had proposed to upstage the N classification of ESCC in the presence of this pathological feature (13, 22). In our study, the construction of various predictive models with modified pathological stage, N parameter, or T parameter was carried out to explore the role of LVI as an upstaging indicator. We further computed the NRI and IDI for all modified staging systems, with original TNM system (Model I) serving as the control to further quantify the additional survival-prediction value attributed to LVI. As demonstrated, Model III, Model IV, and Model IV did not achieve significant improvement in the prediction of survival outcome, while Model II had the best and significant performance, which was internally validated by 1000-bootstrap resampling (**Table 4**, **Supplementary Table S2**). These results support the hypothesis that LVI increases the risk of both lymphatic and hematogenous metastasis, and even represents a more aggressive histological subtype with unknown molecular mechanisms. Therefore, direct upstaging or integration of LVI as a supplementary factor to N or T classification is not recommended in purpose of tailoring treatment strategies. However, it might be a simple way for coarse prognostication and to identify patients at elevated risk of micrometastases. Besides, the presence of LVI may also guide the clinical practice of blood test for minimal residual disease (MRD).

In the past two decades, dozens of studies regarding LVI have been conducted in esophageal cancers (13, 21–24, 26, 33, 34), and the prognostic significance of LVI in LNM-negative or superficial esophageal cancers has been well recognized. Nonetheless, only a few studies have examined its prognostic value in LNM-positive or higher staged esophageal cancers, and these have offered controversial and inconclusive results (13, 21, 22). Moreover, patients in previous studies were mostly staged by an older version of TNM staging system (13, 22). To obtain a more accurate result, our study was conducted on a large sample of ESCC patients restaged by the newest TNM system. An adverse impact of LVI was found in patients with positive node or higher stage diseases, which indicates that a closer surveillance of cancer recurrence is warranted.

LVI was found to be associated with occult lymph node metastasis in various solid tumors including esophageal cancers (29, 32, 35, 36), and therefore, LVI-positive patients might benefit from a multidisciplinary treatment (15, 20). Data on neoadjuvant and adjuvant therapies were presented in our study to address concerns about patient heterogeneity and to provide an insight into real-world practice. Although neoadjuvant chemoradiation followed by surgical resection is the standard treatment for patients with locally advanced ESCC, the number of patients with neoadjuvant therapies and positive LVI was regretfully small to allow further subgroup analysis by TNM stage, nodal status or invasive depth.

Despite the impressive prognostic effects in our study, the incorporation of LVI into the TNM staging system is more for prognostication rather than for decision-making at the current stage. To the best of our knowledge, no prospective studies have been conducted to validate LVI’s practical value. Well-designed prospective trials are warranted to advance the clinical application of LVI. Furthermore, clarifying the molecular mechanisms of LVI may help to identify potential targets for comprehensive treatment (37–42).

Our study also has several limitations. First, this was a retrospective study on a single-institution database. However, this database has been prospectively maintained. The consistency of the institutional procedures of surgery, pathological diagnosis and patient managements could also reduce potential confounding effects. Second, the administration of adjuvant therapies was not balanced between LVI-positive and LVI-negative groups in our study, which might have further complicated the heterogeneity of patients and influence the survival outcomes. Additionally, the number of stage I and pT1 ESCC patients was small in this study, and these results should be interpreted cautiously.

In conclusion, LVI can help with further survival stratification and increase the accuracy of prognostic prediction of the current TNM staging system. Incorporation of LVI as an independent factor into the staging system should be considered and validated by prospective multi-center clinical trials.

## Data availability statement

The raw data supporting the conclusions of this article can be shared per specific institutional review board (IRB) requirements. Upon reasonable request to each respective author/institution, a data sharing agreement can be initiated between the interested parties and the clinical institution following institution-specific guidelines.

## Ethics statement

The studies involving human participants were reviewed and approved by Institutional Review Board of Guangdong Provincial People’s Hospital. Written informed consent for participation was not required for this study in accordance with the national legislation and the institutional requirements.

## Author contributions

I) Study design: WZ, GQ, YT (II) Data collection: WZ, HW, SH (III) Statistical analysis: WZ, YT(IV) Data interpretation: WZ, RC, XB (V)Literature search: WZ, ZZ, JW (VI) Manuscript writing: All authors (VII) Final approval of manuscript: All authors.

## Funding

This study was funded by the Science and Technology Program of Guangzhou, China (202206010103); and Natural Science Foundation of Guangdong Province (2022A1515012469). The funders had no role in study design, data collection and analysis, decision to publish, or preparation of the manuscript.

## Conflict of interest

The authors declare that the research was conducted in the absence of any commercial or financial relationships that could be construed as a potential conflict of interest.

## Publisher’s note

All claims expressed in this article are solely those of the authors and do not necessarily represent those of their affiliated organizations, or those of the publisher, the editors and the reviewers. Any product that may be evaluated in this article, or claim that may be made by its manufacturer, is not guaranteed or endorsed by the publisher.
