# Supplementary material for: The staging performance of a modified tumor-node-metastasis staging system incorporated with lymphovascular invasion in patients with esophageal squamous cell carcinoma
Source: Front Oncol. 2022 Oct 13;12:1018827. doi: 10.3389/fonc.2022.1018827 (PMC9608179; doi:10.3389/fonc.2022.1018827)
Supplement: Supplementary file 1 [file DataSheet_1.docx]

**Supplementary Materials**


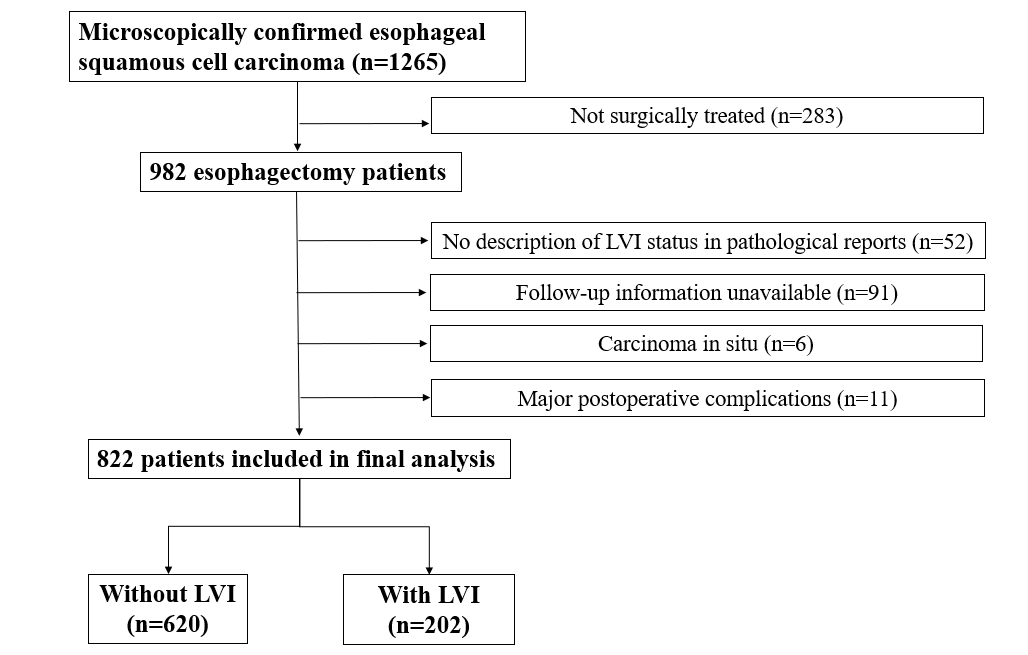


**Supplementary Figure S1** Diagram of patient selection. LVI, lymphovascular invasion.


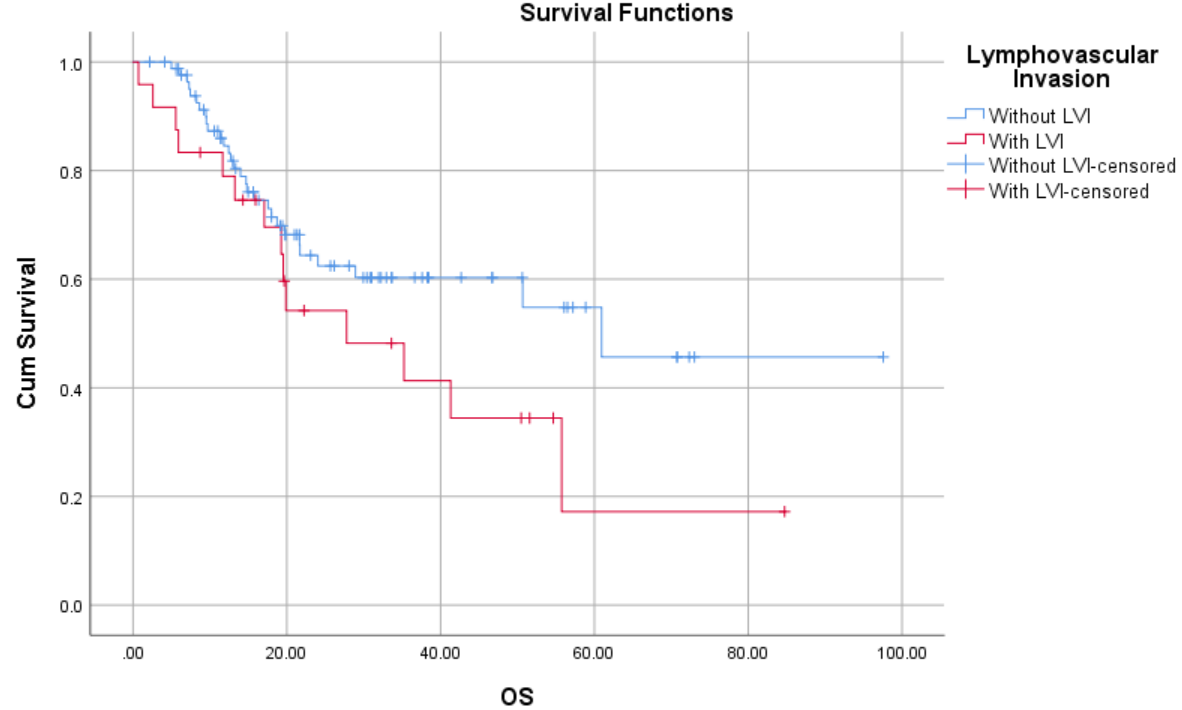


**Supplementary Figure S2** Survival curves for ESCC patients receiving neoadjuvant therapies.

**Supplementary Table S1.** Univariate analysis for overall survival in patients with resected esophageal squamous cell carcinoma

| **Variable (reference)** | **Hazard ratio** | **95% Confidence Interval** | | **p value*** |
| --- | --- | --- | --- | --- |
|  |  | **Lower limit** | **Upper limit** |  |
| **Age, years (<60)** |  |  |  | 0.402 |
| ≥60 | 1.094 | 0.886 | 1.351 |  |
| **Sex (Male)** |  |  |  | **0.049** |
| Female | 0.759 | 0.574 | 1.002 |  |
| **BMI, kg/m^2^ (<18.5)** |  |  |  | **0.128** |
| 18.5-23.9 | 0.854 | 0.625 | 1.169 |  |
| >23.9 | 0.715 | 0.510 | 1.005 |  |
| **CCI (0–1)** |  |  |  | 0.226 |
| ≥2 | 1.291 | 0.851 | 1.958 |  |
| **Tumor Location (Upper)** |  |  |  | 0.692 |
| Middle | 0.876 | 0.630 | 1.218 |  |
| Lower | 0.855 | 0.582 | 1.258 |  |
| **Pathological Stage (IA+IB)** |  |  |  | < 0.001 |
| IIA+IIB | 2.284 | 1.360 | 3.836 |  |
| IIIA+IIIB | 3.562 | 2.123 | 5.977 |  |
| IVA | 5.051 | 2.891 | 8.826 |  |
| **Pathological T stage (pT1a+1b)** |  |  |  | **< 0.001** |
| pT2 | 1.576 | 0.944 | 2.630 |  |
| pT3 | 2.832 | 1.777 | 4.512 |  |
| pT4a+4b | 4.022 | 1.501 | 10.777 |  |
| **Pathological N stage (pN0)** |  |  |  | **< 0.001** |
| pN1 | 1.759 | 1.353 | 2.287 |  |
| pN2 | 1.730 | 1.272 | 2.352 |  |
| pN3 | 2.663 | 1.942 | 3.652 |  |
| **Tumor Grade (G1)** |  |  |  | **0.062** |
| G2 | 1.276 | 0.908 | 1.792 |  |
| G3 | 1.555 | 1.063 | 2.274 |  |
| **PNI (No)** |  |  |  | **0.001** |
| Yes | 1.430 | 1.150 | 1.778 |  |
| **LVI (No)** |  |  |  | **< 0.001** |
| Yes | 1.931 | 1.547 | 2.410 |  |
| **Surgical approach (**Thoracotomy**)** |  |  |  | 0.404 |
| Ivor Lewis | 1.266 | 0.840 | 1.908 |  |
| McKeown | 1.050 | 0.751 | 1.467 |  |
| **Neoadjuvant therapy (No/Unknown)** |  |  |  | **0.019** |
| Yes | 1.470 | 1.066 | 2.029 |  |
| **Adjuvant therapy (No/Unknown)** |  |  |  | **0.005** |
| Yes | 1.352 | 1.095 | 1.670 |  |

95% CI, 95% confidence interval; Ref., reference; BMI, body-mass index; CCI, Charlson comorbidity index; LVI, lymphovascular invasion; PNI, perineural invasion. *Log-rank test. Variables with p value in **bold face** were included in multivariate analysis.

**Supplementary Table S2.** Stability validation of Cox regreesion models using 1000-bootstrap resamples.

| **Models** | **Variables** | **B** | **Bias** | **SE** | **Sig.(2-tailed)** | **95% CI** | |
| --- | --- | --- | --- | --- | --- | --- | --- |
|  |  |  |  |  |  | **Lower** | **Upper** |
| G+T+N+LVI classification | G | 0.185 | 0.005 | 0.098 | 0.055 | 0.004 | 0.381 |
|  | T | 0.428 | 0.001 | 0.098 | 0.001 | 0.247 | 0.635 |
|  | N | 0.192 | 0.003 | 0.056 | 0.001 | 0.086 | 0.308 |
|  | LVI | 0.433 | 0.001 | 0.123 | 0.001 | 0.199 | 0.661 |

T, tumor; N, node; G, grade; LVI, Lymphovascular invasion; B, regression coefficient; SE, standard error; Sig., signigicance; 95% CI, 95% confidence interval
